# Health-Seeking Behavior and Economic Burden of Patients With Type 2 Diabetes Mellitus: A Cross-Sectional Study

**DOI:** 10.7759/cureus.70806

**Published:** 2024-10-04

**Authors:** Bibhash Datta, Bhawna C Datta, Shubhra Dubey, Abhijit Das, Ravindra Manohar, Dharmendra Mandarwal

**Affiliations:** 1 Department of Community Medicine, Tripura Medical College and Dr. BRAM Teaching Hospital, Agartala, IND; 2 Department of Occupational Therapy, Shantha College of Allied Health Sciences, Bangalore, IND; 3 Department of Community Medicine, Government Medical College Satna, Satna, IND; 4 Department of Community Medicine, National Institute of Medical Sciences and Research, Jaipur, IND

**Keywords:** diabetes mellitus, diabetes mellitus type 2, economic burden, health-seeking behavior, india

## Abstract

Background

Type 2 diabetes mellitus (T2DM) is a major public health concern, affecting millions worldwide and placing a significant burden. This study aimed to assess the health-seeking behaviors and the economic burden of T2DM patients in Jaipur, Rajasthan.

Methodology

This cross-sectional observational study was conducted from January 2019 to June 2020 at the Community Health Centre (CHC) in Jaipur, India. A total of 400 T2DM patients, aged 18 years or older and diagnosed for at least six months, were included in the study. Data were collected through a semi-structured questionnaire, focusing on sociodemographic characteristics, health-seeking behaviors, and the economic impact of T2DM. IBM SPSS Statistics for Windows, Version 23 (Released 2015; IBM Corp., Armonk, New York, United States) was used for data analysis.

Results

The study revealed that 50.5% of the participants sought treatment from government hospitals, while 28.7% preferred private healthcare. Allopathic medicine was the most common treatment approach (96.5%). Affordability was the main factor influencing healthcare choices (57.0%). The average annual expenditure on diabetes care was Indian rupees (INR) 15,204, with patients spending INR 1,267 monthly on treatment. About 73.5% of the participants spent between INR 10,001 and 20,000 annually on diabetes care, and 39.8% of patients allocated 0-10% of their monthly income to healthcare expenses.

Conclusion

The results suggest that affordability plays a crucial role in determining health-seeking behaviors. The findings call for policy interventions to improve access to affordable care and mitigate the financial impact of diabetes. Further research is recommended to explore the long-term economic consequences of managing T2DM in similar settings.

## Introduction

Diabetes mellitus is a chronic metabolic disorder marked by persistent hyperglycemia, with type 2 diabetes mellitus (T2DM) comprising approximately 90% of all diabetes cases [1]. Diabetes prevalence is rising more rapidly in low- and middle-income countries than in high-income ones. In 2019, it caused 1.5 million deaths, with 48% occurring before the age of 70 [2]. India ranks second globally in the number of diabetic patients, with 74.9 million individuals aged 20-79 years living with diabetes in 2021, a figure projected to rise to 124.9 million by 2045 [3].

Health-seeking behavior is defined as any action or inaction taken by individuals who perceive a health issue or illness with the intent of finding an appropriate remedy [4]. It is well-established that health-seeking behavior is significantly influenced by the manifestation of symptoms. In addition, a patient’s positive attitude toward their condition, adherence to appropriate health-seeking behaviors, and engagement in necessary self-care practices are crucial in preventing complications [5,6]. Many patients with diabetes are asymptomatic at the time of initial diagnosis, often leading to a lack of urgency in seeking healthcare and contributing to disease denial - a significant barrier to effective disease management [7,8]. There is a tendency among these patients to delay or neglect healthcare measures until complications arise. However, proactive health-seeking behaviors, including early symptom recognition, timely healthcare access, and adherence to treatment, can enhance diabetes control, reduce complication rates, and ultimately improve the quality of life for patients with diabetes [9].

The economic impact of diabetes extends beyond direct medical costs, including hospitalization, medication, and routine care, to encompass indirect costs such as lost productivity, premature mortality, and long-term disability. These financial pressures are particularly challenging in India, where out-of-pocket expenditures constitute a significant portion of healthcare spending, often leading to catastrophic health expenditures and impoverishment [10,11]. Understanding these economic implications is crucial for developing effective health policies and interventions aimed at mitigating the financial impact on patients and the healthcare system.

Although numerous studies have explored the health-seeking behaviors of diabetic patients and the economic burden of managing the disease, both internationally and in various regions of India, no such research has been conducted specifically in Jaipur or in the state of Rajasthan. This study aimed to explore the health-seeking behaviors and assess the economic impact of managing type 2 diabetes among affected individuals in Jaipur.

## Materials and methods

Study design, duration, and setting

This observational cross-sectional study was conducted from January 2019 to June 2020 at the Community Health Centre (CHC) in Jaipur, India. The CHC serves as a field practice area for the Rural Health Training Centre (RHTC) affiliated with the National Institute of Medical Science and Research, Jaipur, India.

Study population and selection criteria

The study population consisted of T2DM patients. All patients diagnosed with T2DM for at least one year and aged 18 years or older were included in the study. Pregnant women, cognitively impaired, critically ill patients, and those who did not provide informed consent were excluded from the study.

Sample size and sampling method

The sample size was determined using the formula \[n = \frac{Z^2pq}{d^2}\]where p = 29.4%, reflecting the proportion of patients who sought healthcare from government health facilities in a previous study [12]. Assuming a 95% confidence interval, 5% precision, and accounting for a 20% non-response rate, the minimum required sample size was calculated to be 400 participants. Non-probability sampling was employed until the required sample size was achieved.

Data collection, processing, and analysis

A pre-designed, pre-tested, and semi-structured questionnaire was used for collecting data by the personal interview method. The questionnaire included questions on sociodemographic characteristics of the study subjects, health-seeking behavior, and economic burden. The direct and indirect costs of diabetic care for the last year were collected in Indian rupees (INR). Collected data were entered into a Microsoft Excel spreadsheet, and subsequently, it was analyzed using IBM SPSS Statistics for Windows, Version 23 (Released 2015; IBM Corp., Armonk, New York, United States). Categorical variables were described using frequency and percentage.

Ethical considerations

This study was approved by the Institutional Ethics Committee of the National Institute of Medical Sciences and Research, Jaipur (NIMSUNI/IEC/2019/41). Participants were explained about the study’s purpose, and written consent was obtained from the participants. The utmost care was taken to maintain the confidentiality and anonymity of the candidates throughout the entire process, including data collection, entry, and analysis.

## Results

Baseline characteristics of study participants

The majority of participants were within the 46-60 year age group. Most participants were male, identified as Hindu, belonged to the Class II socioeconomic group, and had been living with diabetes for the past 1-5 years. Other sociodemographic characteristics of the participants are detailed in Table 1.

**Table 1:** Sociodemographic characteristics of the participants (n = 400) ^#^Modified BG Prasad Classification-2019

Variables	Sub-group	Frequency	Percentage
Age group (in years)	30-45	52	13.0%
	46-60	189	47.3%
	>60	159	39.8%
Gender	Male	239	59.8%
	Female	161	40.3%
Religion	Hindu	392	98.0%
	Others	8	2.0%
Marital status	Unmarried	7	1.8%
	Married	379	94.8%
	Widowed	14	3.5%
Occupation	Unemployed	31	7.8%
	Unskilled	173	43.3%
	Semiskilled	93	23.3%
	Skilled	31	7.8%
	Retired	72	18.0%
Socioeconomic classification^#^	Class I	130	32.5%
	Class II	155	38.8%
	Class III	59	14.8%
	Class IV	39	9.8%
	Class V	17	4.3%
Body mass index	Underweight	32	8.0%
	Normal	108	27.0%
	Overweight	189	47.3%
	Obese	71	17.8%
Duration of diabetes (in years)	1-5	360	90.0%
	>5	40	10.0%

**Figure 1:**
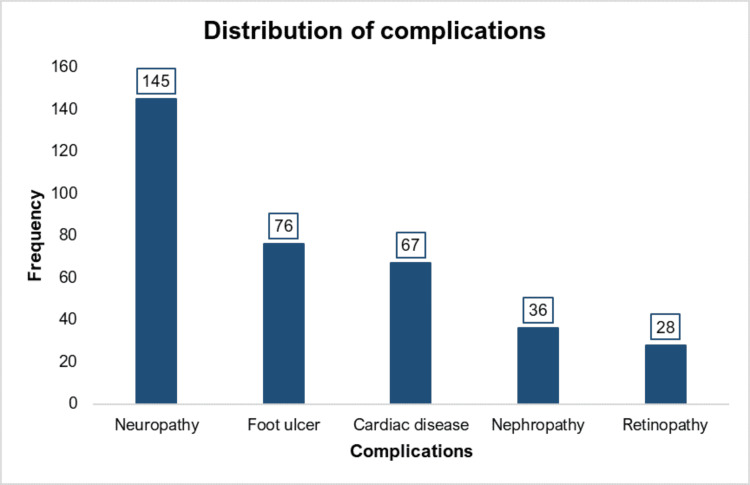
Distributions of complications among participants (n = 400)

More than one-third (36.3%, 145/400) of participants had any complications. Details of complications are depicted in Figure 1.

Health-seeking behavior

The study revealed that 50.5% of patients initially sought treatment at government hospitals, with 28.7% choosing private hospitals. Most patients (88.7%) sought treatment in the early phase of symptom development, while 8.8% did so immediately after diagnosis, and 2.5% waited until symptoms worsened or complications arose. Allopathy was the preferred health system for 96.5% of patients, with the rest 3.5% choosing Ayurveda, Yoga and Naturopathy, Unani, Siddha, Sowa Rigpa, and Homeopathy (AYUSH). All the patients taking allopathic medication reported using oral hypoglycemic drugs, with none of them using insulin. Almost one-third of the patients (33.7%) engaged in self-monitoring of glucose. Regular medication adherence was reported by 94.3% of patients. Government hospitals were the most frequently (57.3%) visited for regular treatment and investigation purposes, followed by private hospitals (23.8%) and private practitioners (17.8%). Affordability was the main reason for choosing a particular health facility (57.0%), followed by accessibility (22.3%), availability (13.7%), and family tradition (7.0%) (Table 2).

**Table 2:** Health-seeking behavior of participants (n = 400) AYUSH: Ayurveda, Yoga and Naturopathy, Unani, Siddha, Sowa Rigpa, and Homeopathy

Parameters	Frequency	Percentage
Place where first treatment sought		
Government hospital	202	50.5%
Private hospital	115	28.7%
Private practitioner	72	18.0%
Chemists	8	2.0%
Home remedies	3	0.8%
Time of seeking treatment		
Just after knowing about the disease	35	8.8%
Early phase when some symptoms developed	355	88.7%
Later when exacerbation of symptoms or complication	10	2.5%
Preference for health system		
Allopathy	386	96.5%
AYUSH	14	3.5%
Keeping allopathic medications for home use		
Yes	386	96.5%
No	14	3.5%
Self-monitoring of glucose		
Yes	135	33.7%
No	265	66.3%
Take medication regularly		
Yes	377	94.3%
No	23	5.7%
Type of healthcare facility visits regularly to receive care		
Government hospital	229	57.3%
Private hospital	95	23.8%
Private practitioner	71	17.8%
Chemists	5	1.3%
Distance of health facility from residence (in kilometer)		
<5	258	64.5%
5-10	116	29.0%
>10	26	6.5%
Reason for preferring particular health facility		
Accessibility	89	22.3%
Affordability	228	57.0%
Availability	55	13.7%
Family tradition	28	7.0%
Visited the health facility for follow-up last year		
<5 times	204	51.0%
≥5 times	196	49.0%

Economic burden of diabetes

The study assessed the annual cost of diabetes care for participants, revealing an average expenditure of INR 15,204 (±7,588) over the past year. This total expenditure included direct costs, such as expenses for drugs, consultations, and travel, as well as indirect costs like lost wages due to hospital stays. On average, monthly spending on diabetes was INR 1,267 (±652), with INR 972 attributed to direct costs and INR 295 to indirect costs. Additionally, it showed that most patients (73.5%) spent between INR 10,001 and 20,000 annually on diabetic healthcare, while 24.0% spent less than INR 10,000 and a small percentage (2.5%) spent over INR 20,000 annually. Regarding the proportion of monthly income allocated to diabetic care expenses, 39.8% of patients spent 0-10% of their monthly income, 33.3% spent 11-20%, 12.5% allocated 21-30%, 10.8% spent 31-40%, and 3.8% spent more than 40% of their monthly income on healthcare (Table 3).

**Table 3:** Distribution of expenditure for diabetic care (n = 400) INR: Indian rupees

Parameters	Frequency	Percentage
Total expenditure (INR/per year)		
<10000	96	24.0%
10001-20000	294	73.5%
>20000	10	2.5%
Proportion of monthly income as expenditure		
0-10%	159	39.8%
11-20%	133	33.3%
21-30%	50	12.5%
31-40%	43	10.8%
>40%	15	3.8%

## Discussion

This cross-sectional study aimed to investigate the health-seeking behaviors of patients with T2DM and assess the economic burden of managing the disease in the rural population of Jaipur, India. The majority of participants were over the age of 45, consistent with existing literature indicating that T2DM is most prevalent in individuals over 45, with the risk increasing with age [1,13,14].

Health-seeking behavior

The study found that 50.5% of patients initially sought treatment at government hospitals, with 28.7% opting for private hospitals. Government hospitals were the most frequently used for regular treatment and follow-up (57.3%), followed by private hospitals (23.8%) and private doctor’s chambers (15.0%). In contrast, a study from urban Pondicherry reported 86.2% of respondents seeking care at public health facilities, while Sekhar and Sekhar found that 80% of participants used government services [12,15]. However, Bhosale et al. observed only 34.1% use public facilities, highlighting regional differences [16]. While Kishore et al. noted a balanced use of both public and private care [17]. In our study, affordability was the primary factor in choosing a health facility (57.0%), followed by accessibility (22.3%), availability (13.7%), and family tradition (7.0%). These findings emphasize challenges in healthcare access, particularly in resource-constrained settings, where public hospitals may be more affordable but limited in services. Private hospitals, while less affordable, could offer better accessibility and perceived care quality. The variation across studies underscores the influence of economic, geographic, and cultural factors on healthcare choices, highlighting the need for policies that improve access to affordable, quality care across different regions. In this study, it was observed that 96.5% of patients preferred allopathy, with only 3.5% opting for AYUSH. This trend aligns with findings by Kishore et al. and Nimesh et al., who observed that patients often navigate between both systems of care, frequently switching providers [7,17]. Self-monitoring of blood glucose was practiced by 33.7% of patients, a figure that contrasts sharply with Kishore et al., who reported no self-monitoring [17]. This discrepancy may reflect differing levels of awareness, education, or access to monitoring tools across populations. Additionally, 94.3% of patients in this study adhered to regular medication, higher than the 82.3% reported by Bhosale et al. and slightly above what Vaishnavi et al. (92.7%) reported [12,16]. However, these figures warrant scrutiny, as self-reported adherence is often subject to recall bias or social desirability, potentially inflating actual adherence rates. Further, there is a need for standardized measures to assess adherence and glucose monitoring across studies.

Economic burden of diabetes

The annual cost of diabetes care among the participants was INR 15,204 (±7,588) over the past year, including both direct and indirect costs. Monthly, participants spent an average of INR 1,267 (±652), with direct costs such as drugs, consultations, and travel accounting for INR 972, and indirect costs like lost wages during hospital stays amounting to INR 295. The distribution of annual spending revealed that the majority (73.5%) of patients spent between INR 10,001 and 20,000, and only 2.5% spent over INR 20,000. The expenditure on diabetes care varies across the literature. For instance, Prajapati et al., where the mean total cost was INR 12,391.84, with contributions from direct medical costs, direct non-medical costs, and indirect costs being 74%, 2%, and 24%, respectively [18]. Thakur et al. found a mean annual expenditure of INR 8,958 (±11,704), with 14% of subjects spending more than INR 20,000 annually [19]. Similarly, Nagarathna et al. reported an annual expenditure of INR 13,179 per capita, with about 17% of household expenditure devoted to healthcare [20]. Regarding the proportion of monthly income allocated to diabetic care expenses, the majority (39.8%) of patients spent 0-10% of their monthly income, while 3.8% spent more than 40% of their monthly income on diabetic care. Thakur et al. reported that the majority of subjects (66.7%) spent <5% of their family income on diabetes care. However, 9.3% of participants spent more than 20% of their family income on diabetes care and all of them belonged to the socioeconomically weaker sections [19].

While the reported expenditures in this study align with previous research, several critical points require consideration. First, the wide standard deviations across studies, particularly in Thakur et al. (±11,704 INR) and even in the current study (±7,588 INR), highlight substantial variability in healthcare costs among individuals, suggesting that certain groups may bear a disproportionate financial burden [19]. This variability could stem from differences in access to healthcare services, regional disparities, or variations in disease severity, which are not sufficiently addressed in the studies. Moreover, most participants in this study spent between INR 10,001 and 20,000, indicating a substantial financial commitment for most households, which may limit access to care for those with lower socioeconomic status.

Strengths and limitations

Strengths of this study include being the first of its kind conducted in rural Rajasthan to explore health-seeking behavior and the financial burden associated with managing T2DM. Additionally, the study benefits from a large sample size, which enhances the strength of the findings. However, there are potential limitations. Since data were collected exclusively from participants who visited the rural healthcare facility, the findings may not be generalizable to the wider population.

## Conclusions

This study reveals major gaps in diabetes care access and affordability in rural Rajasthan. While most patients sought early treatment at government facilities for cost reasons, many still face a heavy financial burden, with nearly one-third spending over 20% of their income on diabetes management. Despite good medication adherence, these expenses highlight the need for more affordable and accessible care. Public health strategies should focus on reducing costs, improving drug availability, and expanding health education at primary health centers. Future research, preferably qualitative research, should examine long-term economic effects and ways to reduce healthcare disparities in rural and low-income areas.

## Appendices

Study title: Health-Seeking Behavior and Economic Burden of Patients With Type 2 Diabetes Mellitus: A Cross-Sectional Study

Participants ID…………………                                               Date of Interview ……/……/20…..

I. Socio-demographic details & Diabetic characteristics

1. Reg. No: .............. 

2. Name: .................................................................                

3. Age (in years): ...........                                                                4. Gender: M/F

5. Religion: Hindu/Muslim/Christian/Jain                               

6. Address: ………………….......................................................................................................       

7. Height (in cm): …...............................                                        8. Weight (in Kg): …………

9. Marital status: Unmarried/Married/Widower/Widow             

10. Family type: Nuclear/ Joint

11. Total Family members:

12. Annual Family Income (in INR): ………………………..

13. Occupation: …………………..

14. Diabetic for last: 1-5 year / >5 years

15. What treatment you are taking currently?

No treatment / Diet / OHA / Insulin

16. Complication: Yes/ No

If yes, please specify……………………………………………………………

III. Health seeking behaviour

17. Where did you seek treatment for the first time for diabetes?

Govt Hospital/Private Hospital/ Private practitioner/Dispensary/From Chemists/Home Remedies

18. How long after the diagnosis of the disease, did you seek treatment?

Just after knowing about the disease/When the disease has extended to some extent/Later stage when exacerbation of symptoms or complication

19. Which health system do you prefer?                                     Allopathy/AYUSH

20. Do you keep any allopathic medicine for home use?              Yes/No

21. Do you monitor glucose by yourself at home?                       Yes/No

22. Do you take diabetic medicine regularly?                              Yes/No

23. Which type of healthcare facility do you visit regularly to receive care?

Govt Hospital/Private Hospital/ Private practitioner/Dispensary/Chemists

24. How far is your preferred health center situated from your house?

Within 5 km/5-10 km/ >10 km

25. What is the main reason that you go to that particular facility?

Accessibility/Affordability/Availability/Family tradition

26. In the last year, how many times did you visit the facility for follow-up?........

III. Economic burden

27. Kindly tell me the cost (in INR) incurred for the following in last year

a)     Travel cost

b)     Consultation Fee

c)     Cost of Drugs

d)     Cost of Laboratory Investigations

e)     Cost of Attendant

f)      Hospital Cost

g)     Self-monitoring Cost

h)     Cost of Treating Complications

i)       Loss of wages due to hospital stays

Total Cost [add (a) to (i)] ………………….

28. Total direct cost (in INR) …..………….

29. Total indirect cost (in INR) ……………..

30. Proportion of monthly income as diabetic expenditure

 0-10%/11-20%/21-30%/31-40%/41-50%/>50%
